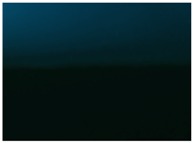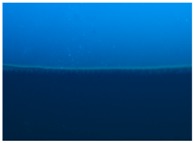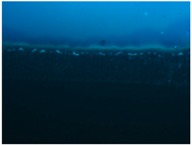# The Influence of Biochemical Modification on the Properties of Adhesive Compounds

**DOI:** 10.3390/polym9010009

**Published:** 2016-12-31

**Authors:** Anna Rudawska, Izabela Haniecka, Magdalena Jaszek, Monika Osińska-Jaroszuk

**Affiliations:** 1 Faculty of Mechanical Engineering, Lublin University of Technology, 20-618 Lublin, Poland; i.haniecka@wp.pl; 2 Department of Biochemistry, Maria Sklodowska-Curie University, 20-033 Lublin, Poland; magdalena.jaszek@poczta.umcs.lublin.pl (M.J.); monika.osinska-jaroszuk@poczta.umcs.lublin.pl (M.O.-J.)

**Keywords:** epoxy adhesive, biochemical modification, properties

## Abstract

The main objective of this study was to determine the effect of biochemical modification of epoxy adhesive compounds on the mechanical properties of a cured adhesive exposed to various climatic factors. The epoxy adhesive was modified by lyophilized fungal metabolites and prepared by three methods. Additionally, the adhesive compound specimens were seasoned for two months at a temperature of 50 °C and 50% humidity in a climate test chamber, Espec SH 661. The tensile strength tests of the adhesive compounds were performed using a Zwick/Roell Z150 testing machine in compliance with the DIN EN ISO 527-1 standard. The examination of the adhesive specimens was performed using two microscopes: a LEO 912AB transmission electron microscope equipped with Quantax 200 for EDS X-ray spectroscopy and a Zeiss 510 META confocal microscope coupled to an AxioVert 200M. The experiments involved the use of a CT Skyscan 1172 tomograph. The results revealed that some mechanical properties of the modified adhesives were significantly affected by both the method of preparation of the adhesive compound and the content of the modifying agent. In addition, it was found that seasoning of the modified adhesives does not lead to a decrease in some of their mechanical properties.

## 1. Introduction

The available literature provides information about the methods for modifying adhesive compounds [1,2,3,4,5,6] to obtain specific properties [7,8,9,10,11,12] and improve their adhesive properties (which are vital for producing adhesive joints) [7], e.g., by the addition of different modifiers such as nanofillers [13,14,15,16] and lignin fillers [17,18,19,20,21,22,23,24,25,26]. Ghaffar and Fan [17] provided an overview of the problem, focusing on the chemical structure and composition of lignin in straw as well as the modification and application thereof as an adhesive.

Wood-decaying fungi, particularly white rot fungi, have attracted the interest of numerous researchers due to their remarkably effective biodegradation system [27,28,29,30,31]. Given their effective production of diverse secondary metabolites, e.g., enzymes such as laccase, which take part in the degradation of plant biopolymers, transformation processes for various kinds of aromatic derivatives, paper pulp bleaching, and the decomposition of dyes or removal of environment pollutants, these organisms have long been used in different areas of biotechnology [19]. Laccase is also used in the research on modification of the adhesive properties of plant biopolymers such as lignin [19,20], which can be found in resins or adhesives for the adhesive bonding of wood [17,22,23,24,25]. Lignin has been used in epoxy resin, and many different formulation approaches have been investigated [26,27,28]. It is known that lignin-based adhesives have a potential for engineering applications due to their environmental suitability as well as economic and technical feasibility [17,20].

Wood-decaying fungi are also a source of very important bioactive metabolites, such as polysaccharides, and a wide range of low molecular weight substances (peptides, alkaloids, dyes) with anti-oxidative, antibiotic, and anti-cancer properties [30,31,32,33,34,35]. Given the metabolic biodiversity of these fungi as well as the possibility of reproduction under normalized laboratory conditions, the use of wood-decaying fungi has good prospects in research on adhesive compound modification. Besides production of materials with new properties, this can lead to development of a new application for this group of biomaterials [36,37,38,39,40]. This problem falls in line with the trend of finding new applications for natural bioproducts [17,21,24]. For example, they can be used in processes related to environmental protection, natural dye synthesis, modification of composite materials (e.g., using bone-substituting fungal glucans), or in various medical applications (polysaccharide nanomolecules, natural antibiotics, and cytostatics) [33,34]. It is known that a number of organisms in nature, such as fungi and bacteria, are also able to form biofilms on the surface of various types of materials. The first stages of biofilm formation are closely related to the process of adhesion of microorganisms to the surface of a material. The initial reversible adhesion is executed principally by non-specific hydrophobic, electrostatic, and van der Waals interactions. Further, in the course of irreversible adhesion, microorganisms produce organic substances (polysaccharides, proteins, nucleic acids, phospholipids, or surfactants) that promote this process. Therefore, it can be assumed that some of these natural substances can probably be an interesting source of adhesive modifiers. The composition and properties of *Pycnoporus sanquineus*, i.e., a low molecular weight subfraction used as a modifying agent in the present work, are precisely described in [41]. Besides the unique qualitative composition of the fungal preparations used in this work, they also seem noteworthy, especially in the context of modification of adhesive mixtures and due to their very high antioxidant and antibacterial potential.

There are adhesive compounds that contain epoxy resins and curing agents as well as substances modifying the properties of both liquid and solid adhesives [1,4,5,7,12,42]. In addition, there are numerous modifiers for adhesives such as fillers, diluents, thixotropic agents, antistatic agents, elasticizing compounds, and coloring agents. Popular adhesive compounds are based on the use of mineral fillers as modifiers in order to prolong the life of the compounds, improve some of their mechanical properties, and increase their chemical and ageing resistance [1,2,5,7,24]. The most widely used fillers are inorganic substances, usually mineral fillers in the form of quartz powder, graphite, metal powders, cut glass, or metal fibers produced by grinding natural materials. In popular adhesive compounds, the quantity of added fillers can vary, e.g., from 25% to 50% or even up to 95%; in the case of dry organic pigments, it is about 0.5%, while the content of inorganic pigments can range from 2% to 4% [43].

The primary objective of this study is to determine the influence of the biochemical modification of an epoxy adhesive compound on some mechanical properties of a cured adhesive exposed to different climatic factors. Based on the results of preliminary tests, it can be supposed that the use of metabolites derived from fungal cultures as modifiers for epoxy compounds could have a positive effect on not only the strength of adhesive joints exposed to various climatic conditions but also the ageing and degradation processes of the adhesive compounds. Epoxy compounds are used to prepare adhesive joints made of aluminum alloy sheets and galvanic zinc coated sheets, and one of the requirements is to obtain the desired high strength and elastic adhesive layer as well as the increased life of the adhesive joints. In this study, the first step of the research involved examination of the mechanical properties of modified epoxy adhesive compounds. The findings about these properties then served as a basis for development of a technology for making adhesive joints.

## 2. Materials and Methods

### 2.1. Characteristics of Basic Adhesives

The basic adhesive was prepared by mixing Epidian 53 epoxy resin and a polyamide curing agent (PAC) in a 1:1 stoichiometric ratio. The epoxy resin and curing agent were manufactured by Organika-Sarzyna, Nowa Sarzyna, Poland [44,45]. The addition of the curing agent to the epoxy resin initiates the curing process, wherein the adhesive transforms from a liquid state to a hard and resistant solid. During this time, known as compound life (gelation time), two tested adherends must be bonded [4,42,43]. Individual ingredients of the adhesive were weighed using a TP-2/1 scales (manufactured by FAWAG S.A., Lublin, Poland), with the ISO9001 certificate and the measuring accuracy of 0.1 g.

#### 2.1.1. Epoxy Resin

**Figure 1:**
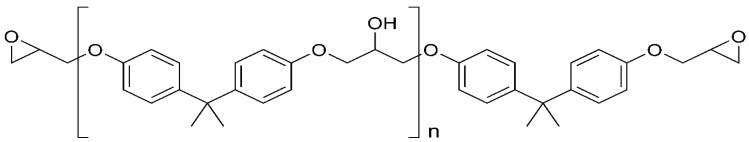
Structure of bisphenol-A diglycidyl ether epoxy resin [46].

Epoxy resins are composed of long chain-like molecules similar to those of vinyl ester, with reactive parts at both ends (Figure 1). The difference is that the reactive parts are composed of epoxy and not ester groups. The absence of ester groups means that epoxies are characterized by a very high level of water resistance. What is more, the epoxy molecule contains two nucleus groups capable of carrying mechanical and thermal loads more efficiently than the straight groups, which contributes to the excellent strength, rigidity, and thermal properties of epoxies.

Epidian 53 is a liquid epoxy compound with colors ranging from yellow to dark–brown and a distinctive odor of aromatic hydrocarbons. It exhibits low viscosity and average reactivity. It is produced by thinning Epidian 5 with styrene in a quantity ranging from 13 ns to 15 ns [43]. Containing the inactive diluent, Epidian 53 has a low viscosity (900–1500 mPas at 25 °C), average reactivity, and high insulation properties. The density of this epoxy resin is 1.11–1.15 g/cm^3^ at 20 °C and the number of epoxy is 0.41 [44]. The curing of an adhesive compound at an elevated temperature significantly accelerates polyreaction. When used as an adhesive, Epidian 53 exhibits the highest adhesive joint shear strength when cured at a temperature of approx. 110 °C and under a pressure amounting even up to 22.5 MPa. The addition of styrene results in reduced viscosity of the compound and, hence, its enhanced processing properties.

#### 2.1.2. Curing Agent

PAC is a modified polyamide curing agent fabricated by the polycondensation of polyamine with dimers of unsaturated fatty acid methyl esters. It is primarily used for modifying and curing low molecular weight epoxy resins and compounds based on these epoxy resins. The addition of PAC results in higher elasticity and increased impact strength of cured compounds. PAC is a viscous brown liquid. At room temperature, the life of a compound containing this curing agent amounts to several hours, while the total cure time is 4–7 days. To accelerate polyreaction, the curing process can be run at a temperature of about 60 °C for 6–8 h. Compounds with high contents of PAC facilitate formation of more elastic plastics with higher impact strength but lower hardness at an elevated temperature, compared to those with other curing agents. PAC-containing adhesives are used for the adhesive bonding of fragile structures prone to significant deformation. Such joints operate well when the temperature is below 0 °C [42,45].

### 2.2. Characteristics of Biochemically Modified Adhesive

A mixture of the resin and the curing agent in a 1:1 ratio (Epidian 53/PAC/1:1) was modified by a biological material, i.e., the lyophilized fungal preparation. Lignin cellulose-degrading *Pycnoporus sanguineus* (L.) *Muller* (Department of Biochemistry, Maria Sklodowska-Curie University, Lublin, Poland) from the phyllum *Basidiomycetes* belonging to white rot fungi (WRF) was used in this study. The modifying agent used in the experiments was an extracellular low molecular-weight secondary metabolite subfraction obtained by FPLC (Fast Protein Liquid Chromatography, BIO-RAD, Hercules, CA, USA). Low molecular weight subfractions with their weight below 10 kDa were then thickened by reverse osmosis and lyophilized. After determination of the biochemical composition as well as the antioxidative and antibiotic properties of this material, the preparation was used for further examination. The lyophilized fungal material (Department of Biochemistry, Maria Sklodowska-Curie University, Lublin, Poland) was added to the adhesive mixtures in the concentrations listed in Table 1. The adhesive compounds were produced according to the methods listed in Table 2 by mechanical mixing with a specially contoured mixer (Faculty of Mechanical Engineering, Lublin University of Technology, Lublin, Poland) operated at the speed of 460 rev/min. The mixing time was set to 2 min. Next, gas cavities were removed from the mixture for 3 min on the test stand.

### 2.3. Shape and Dimensions of the Cured Adhesive Specimens

**Figure 2:**
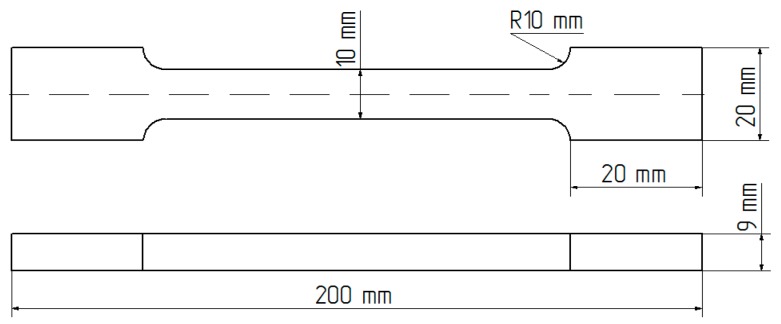
Dimensions of the adhesive specimens used for strength testing.

**Figure 3:**
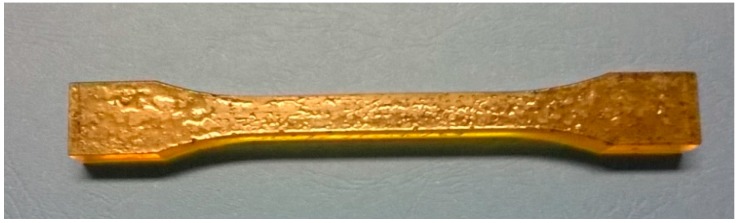
Specimens of the cured adhesive after 7 days of curing.

The shape and dimensions of the test specimens are shown in Figure 2, while Figure 3 shows the adhesive samples after 7 days of curing.

### 2.4. Preparation of the Cured Adhesive

The specimens of the cured adhesive joints were prepared using a silicone mold (Faculty of Mechanical Engineering, Lublin University of Technology, Lublin, Poland) with the required shape. The adhesive compounds were prepared in compliance with the methods described in Section 2.2, and the liquid compound was distributed over the mold by a dosing container (Faculty of Mechanical Engineering, Lublin University of Technology, Lublin, Poland). In addition, POLISILFORM (Polish Silicone, Nowa Sarzyna, Poland) was used to facilitate separation of the produced adhesive compounds from the mold. POLISILFORM is a silicone agent that prevents adhesion of plastics to the mold used for polymer and rubber processing. The use of this substitute of talc increases the life of silicone molds. POLSILFORM is a colorless, odorless, and solvent-free substance. When sprayed, it produces a thin layer of silicone oil with anti-adhesive properties on the mold surface. Prior to use, it must be shaken and then sprayed onto the mold surface from a distance of about 30 cm. The adhesive compounds were prepared under the following conditions: Ambient temperature: 23 ± 2 °C and humidity: 23% ± 2%. The curing process was run in a single stage for 7 days under the same conditions as those applied in the preparation of the compound. The seasoning process was run for two months at a temperature of 50 °C and 50% humidity in an Espec SH 661 climatic test chamber (ESPEC North America, Inc., Hudsonville, MI, USA). The conditions of specimen preparation and seasoning are compared in Table 3.

### 2.5. Strength Tests

Tensile strength tests of the prepared adhesive compounds (after the specified seasoning period—Table 3) were performed using a Zwick/Roell Z150 testing machine (Zwick Roell, Kennesaw, GA, USA) in compliance with DIN EN ISO 527-1. The test speed was set to 2 mm/min. The specimens of the cured adhesive were mounted in the screw-wedge clamps of the testing machine. The tests were performed under the following conditions: ambient temperature: 23 ± 2 °C and humidity: 23% ± 2%.

The strength tests were performed for 10 specimens in 3 test runs per each variant of the adhesive preparation method and seasoning time (Table 3) in 5 test runs depending on the applied adhesive, taking account of the modified and unmodified materials (5 × 10 test runs). The total amount of specimens was 150 items.

### 2.6. Microscopic Examination

Two microscopes were used in the experiments: A LEO 912AB transmission electron microscope (Zeiss, Jena, Germany) equipped with Quantax 200 for energy-dispersive X-ray spectroscopy (EDS) and a Zeiss 510 META confocal microscope (Zeiss, Jena, Germany) coupled with Axiovert 200 M. The inverted microscope Axiovert 200 M is equipped with an LSM 5 Pascal head (with magnification 200×), which facilitates non-invasive visualization of such structures as biofilms, microcapsules, and other film-forming substances as well as crystallization of substances by means of laser fluorescence, standard fluorescence, and polarization contrast. Moreover, it allows geometric and densitometric measurements as well as assessment of the structure edge. In addition, the experiments were conducted with the use of a CT Skyscan 1172 tomograph (Brucker MicroCT, Kontich, Belgium).

## 3. Results

### 3.1. CT Examination of the Structure of the Modified Adhesive

**Figure 4:**
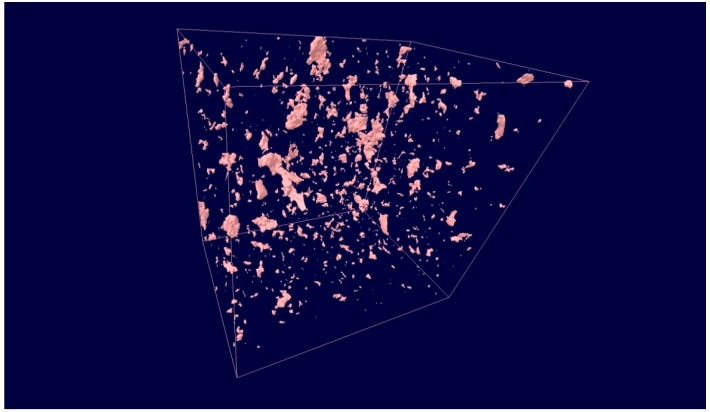
Computer tomography (CT) image of the structure of the adhesive modified with 0.50% of the modifier (lyophilized fungal preparation).

**Figure 5:**
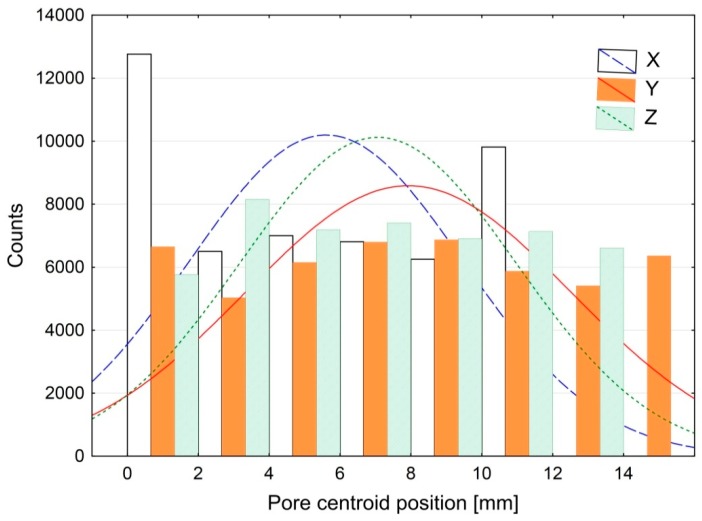
Histogram of the coordinate distribution describing the location of pores in the adhesive modified with 0.50% of the modifier (lyophilized fungal preparation).

The use of computer tomography (CT) allowed visualization of the structure of the modified adhesive containing the lyophilized fungal preparation as a modifying agent. Figure 4 shows a CT image of the structure of the modified adhesive, while Figure 5 provides some information about the tested structure.

The CT results provide information about the structure of the adhesive subjected to biochemical modification. Figure 4 illustrates the distribution of the modifier in the modified adhesive compound, which can serve as preliminary assessment of the applied adhesive preparation method, including the mixing method and technological parameters of the mixing process. The histogram of the coordinate distribution (Figure 5) describing the location of pores in the modified adhesive specimen provides quantitative characteristics of the adhesive.

### 3.2. Strength Test Results—Analysis of the Effect of the Adhesive Compound Preparation Methods on the Mechanical Properties of the Adhesive

The tensile strength tests of the adhesive compounds conducted in compliance with the DIN EN ISO 527-1 standard allowed determination of the following parameters: *F*—tensile force, *E_t_*—tangent modulus, σ_γ_—shear stress, σ_M_—tensile stress, ε_M_—relative tensile strain, and σ_B_—stress at break. The results of the tangent modulus *E_t_* of the modified adhesive compounds produced with Methods I and II (Variants I and II) are given in Table 4.

In Method I, the highest value of *E_t_* was obtained for the adhesive modified with 0.75% of the modifier, amounting to 464 MPa (Table 4). The *E_t_* value of the adhesive modified with two modifier concentrations, 0.50% and 0.75%, was higher than that of the unmodified adhesive. This difference was 2% and 10%, respectively. In the other cases, the modulus of the modified adhesives was lower than that of the unmodified adhesive. In Method II (Table 4), the highest *E_t_* value was obtained for the adhesive modified with the 1.00% modifier concentration, and it amounted to 1024 MPa. The *E_t_* value obtained in the strength tests for the adhesives modified with the 0.50% and 0.75% modifier concentrations was similar to that of the unmodified adhesive. The highest difference was observed for the case with the highest modifier content (1.00%), as it amounted to 19%. In the specimens with 0.25% of the modifier, the static modulus of the modified adhesive was lower than that of the unmodified adhesive.

**Figure 6:**
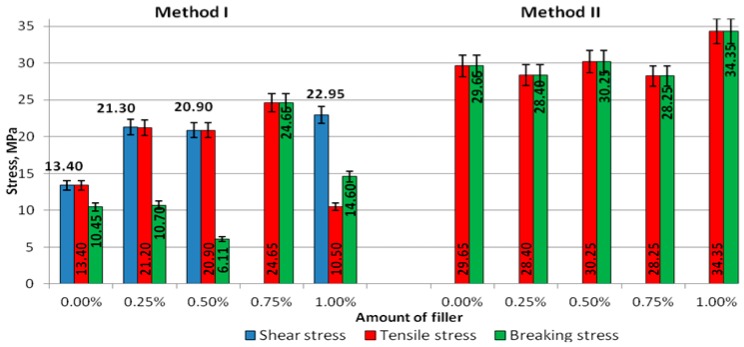
Comparison of the stresses in the adhesives prepared with Methods I and II.

The results of stresses of the cured adhesive specimens prepared according to Methods I and II are shown in Figure 6.

The obtained shear stresses σ_γ_ of the adhesive compounds prepared with Method I at concentrations of 0.25% (21.3 MPa), 0.50% (20.9 MPa), 0.75% (24.7%), and 1.00% (23.0 MPa) were higher than σ_γ_ obtained in the control experiment (0%), i.e., 13.5 MPa (Figure 6). A similar trend was observed for the tensile stress σ_M_: The tensile stresses for the modified adhesive in each variant of modification were higher than those obtained for the unmodified adhesive (13.5 MPa). In Method II, the tensile stresses (σ_M_) and the stresses at break (σ_B_) were lower than those produced in the test with no modifier (σ_M_—29.7 MPa, σ_B_—29.7 MPa) only for the modifier concentration of 0.25% (σ_M_—28.4 MPa, σ_B_—28.4 MPa) and 0.75% (σ_M_—28.3 MPa, σ_B_—28.3 MPa). For the other modified adhesives, the tensile stresses (σ_M_) and stresses at break (σ_B_) were higher than those observed for the unmodified adhesive.

Comparing the stresses in the adhesives prepared with Methods I and II (Figure 6), it can be seen that the tensile stresses (σ_M_) and the stresses at break (σ_B_) were much higher in the adhesive specimens made with Method II and the difference between them amounted almost to 18%. In Method I, the stresses measured in the zero test (σ_γ_—13.4 MPa, σ_M_—13.4 MPa, σ_B_—10.5 MPa) were lower than those produced for the modifier concentration of 0.25% (σ_γ_—21.3 MPa, σ_M_—21.2 MPa, σ_B_—10.7 MPa). Compared to the unmodified adhesive, higher stresses were observed in a majority of cases involving the modified adhesive, although the stress at break in the modified adhesive containing 0.50% of the modifier was much lower than that in the unmodified adhesive. In turn, the adhesive modified with 1.00% of the modifier had a lower value of tensile stress than the unmodified adhesive. Regarding the modified adhesive, the difference between the tensile stresses was 43%, while that between the stresses at break was 75%. It can be claimed that the application of Method I for preparation of the adhesive leads to significant variations in the mechanical properties of cured adhesive compounds.

**Figure 7:**
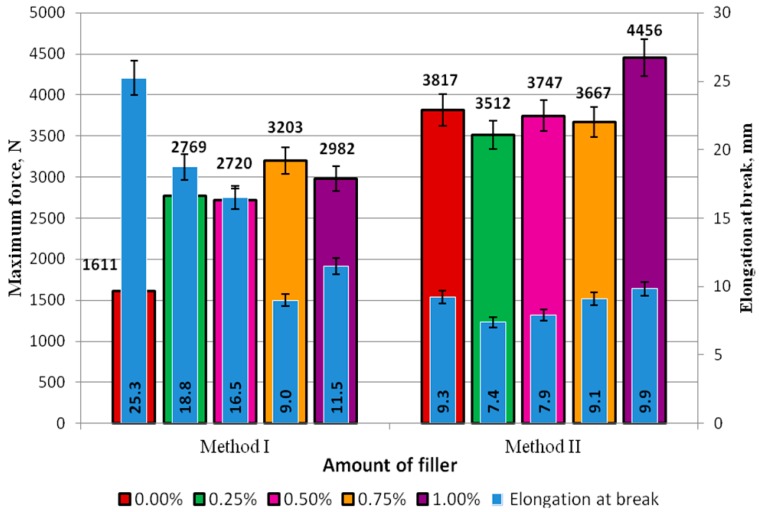
Comparison of the maximum force and elongation at break in the adhesive specimens prepared with Methods I and II.

The standard force and elongation of the adhesive specimens made with Methods I and II are compared in Figure 7.

In Method I for preparation of the adhesive (Figure 7), the highest elongation at break, was produced for the unmodified adhesive and it amounted to 25.3 mm. It was observed that with the increasing modifier concentration in the adhesive compound, the elongation at break decreased. The correlation coefficient of these quantities is 0.85. It can be claimed that an increase in the modifier content leads to a decrease in the elongation and thus reduced elasticity of the adhesive. The difference between the highest (18.8 mm at 0.25%) and the smallest elongation at break (9.0 mm at 0.75%) was 9.8 mm, which almost amounted to 50%.

In Method II for preparation of the adhesive compound (Figure 7), no significant differences were observed with respect to elongation at break. The elongation at break ranged from 7.4 mm (at 0.25% modifier content) to 9.9 mm (at 1.00% modifier content), and the difference between these quantities was 25%. An increase in the elongation at break was observed with the increasing modifier content. The correlation coefficient is 0.95. It can therefore be predicted that an increase in the modifier content (lyophilized fungal preparation) leads to a higher elasticity level of the adhesive. The elongation at break of the unmodified adhesive was smaller only for the adhesive compound with the highest modifier content tested, i.e., 1.00%. This difference amounted to 6%.

In Method I (Figure 7), the maximum force in the control experiment was 1611 N, which was lower than the force obtained for the specimens with concentrations of 0.25% (2769 N), 0.50% (2720 N), 0.75% (3203 N), and 1.00% (2982 N). Regarding the modified adhesive, the highest force, i.e., 3203 N, was obtained for the specimens with 0.75% of the modifier; this value of force was almost half as high as the force obtained for the unmodified adhesive specimens (1611 N). In Method II, only the results of the tests involving the 0.25% and 0.75% modifier concentrations were lower than the results of the maximum force of the referential specimens of the unmodified adhesive (3817 N).

The values of elongation at break were higher for the specimens obtained with Method I, compared to those obtained with Method II; still, among all the modifier concentrations, they were smaller than the results obtained in the 0.00% test (25.3 mm). In Method II, the elongation at break of Epidian 53/PAC with the 1.00% modifier concentration (9.9 mm) was higher by 0.50 mm than that yielded in the control experiment.

The results given in Figure 7 reveal that the failure forces were much higher in the adhesive specimens prepared with Method II, and the difference between these values for the respective modifier concentrations was as follows: 0.25%–21%, 0.50%–28%, 0.75%–13%, and 1.00%–32%. It can be claimed that the application of Method II yielded similar results, although with a smaller scatter of values than in Method I. Based on the results obtained, it seems that the addition of modifiers affects both growth and reduction of various mechanical properties, which is dependent on the method of preparation of the adhesives. In the case of Method II, it can be noted that the increasing level of modifier addition causes an increase in both the breaking force and elongation. The analysis of tensile strength shows that the increasing addition of the modifier results in a significant increase in the strength when Method I is applied and absence of a significant impact in a majority of cases in Method II. The knowledge of these values and relationships will allow a design of adhesive joints in accordance with their intended use. In cases where greater strength is required, an adhesive prepared with Method II can be used, whereas Method I will ensure greater flexibility. Addition of modifiers designed to improve aging resistance is also important.

### 3.3. Strength Test Results—The Effect of Seasoning of the Adhesive Compounds on the Mechanical Properties of the Adhesive

The results of the mechanical properties of the adhesive compounds produced with Method I after the two-month seasoning (Variant III—Table 3) are given in Table 5.

The lowest values of failure force, static modulus, shear stress (σ_y_), tensile stress (σ_M_), and stress at break (σ_B_) were measured for the unmodified adhesive. In the control test, the tensile stresses and the stresses at break had the same value of 8.8 MPa, only exceeding the results obtained in the test with 1% of the modifier. In all tested cases of the modified adhesives (except for the stress at break of the adhesive modified with 1.00% modifier), the mechanical properties of the adhesive specimens subjected to seasoning for 2 months at a temperature of 50 °C and 50% humidity were higher than those noted for the unmodified adhesive. For all the modifier concentrations, the tensile stresses were higher, ranging from 2.1 MPa to 4.4 MPa, while the stresses at break ranged from 1.38 MPa to 2.6 MPa, compared to the control test results (σ_M_—8.8 MPa, σ_B_—8.8 MPa). It can therefore be stated that the addition of the lyophilized fungal preparation has a positive effect on the mechanical properties of the adhesive during the seasoning period.

The highest tensile stress was observed for the adhesive containing 0.75% of the modifier (13.2 MPa); yet, in the other cases, the results do not differ significantly. This value was higher by 33% than that of the referential specimens. As for the modified adhesives, the lowest stresses were obtained for the adhesive with the highest modifier concentration, i.e., 1.00%.

Comparison of the results of tensile strength for the adhesive specimens subjected to climatic chamber seasoning (Variant III, Table 5) with those for the adhesive specimens made with the same method but seasoned at ambient temperature for 7 days (Variant I; Figure 6) revealed that the stresses significantly decrease after the climatic chamber seasoning. The smallest differences were observed with respect to stresses at break. The stress at break in the modified adhesive containing 0.50% of the modifier after the seasoning was higher by over 40% (10.7 MPa) than the stress of break of the adhesive after 7 days of curing (6.1 MPa). The stress at break was also higher for the adhesive modified with 0.25% of the modifier; however, this difference was not significant and only amounted to 6%. For this reason, it can be claimed that the seasoning of the modified adhesives does not lead to reduction of some of their mechanical properties; it was observed in the case of the unmodified adhesive that each of the tested quantities decreased after the seasoning.

**Figure 8:**
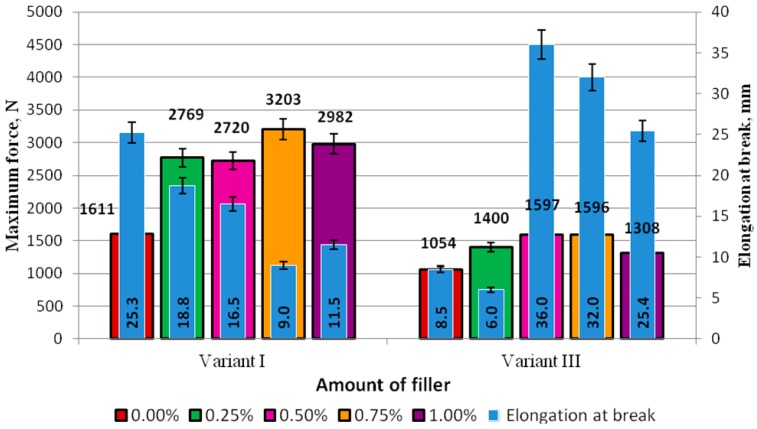
Comparison of the maximum force and elongation in the adhesive specimens prepared according to Method I and subjected to seasoning for 7 days (Variant I) and two months (Variant III).

The standard force and elongation at break of the adhesive specimens prepared according to Method I and subjected to seasoning for 7 days (Variant I) and two months (Variant III) are compared in Figure 8. The diagram in Figure 8 shows that the values of elongation at break of the specimens prepared with the modifier concentrations of 0.50% (36.0 mm), 0.75% (32.5 mm), and 1% (25.4 mm) were not only much higher than the elongation results obtained in the control experiment (8.5 mm) but also the stresses yielded in the experiments. In the control experiments, the stresses were equal to 8.5 MPa and only exceeded the stresses of the specimens with the modifier concentration of 1.00% (7.3 MPa).

The elongation at break of the adhesive specimens modified with 0.50% of the modifier was higher by 0.56% than that of the same adhesive that was not subjected to seasoning. An even more significant difference, i.e., 72%, was observed regarding the elongation of the adhesive with 0.75% of the modifier. The elongation of the adhesive with 1.00% of the modifier after seasoning was higher by 55% than that of the modified adhesive with the same modifier content and cured for 7 days.

An analysis of the results of the failure force demonstrated that this force was smaller for all adhesives subjected to seasoning, and the differences between the values of this parameter were as follows: Unmodified adhesive (0%)—34%, adhesive with 0.25% modifier—41%, adhesive with 0.50% modifier—41%, adhesive with 0.75% modifier—50%, and adhesive with 1.00% modifier—54%. The results demonstrate that the maximum force decreased nearly by half for all modified adhesives compared to the modified adhesives that were not subjected to seasoning. It can therefore be claimed that although seasoning has a negative effect on the failure force of the adhesive, it leads to a higher elasticity level of the adhesive. In the majority of cases, the elongation at break increased by over 50% (and even by over 70%), compared to that yielded for the modified adhesives that were not subjected to seasoning. It was observed that the elongation of the modified adhesives after seasoning even increased by nearly 30% (0.50% modifier) and 27% (0.75% modifier) compared to the elongation results obtained for the unmodified adhesive cured for 7 days at ambient temperature (25.3 mm). These results are similar to those yielded for the adhesive with 1.00% of the modifier. It was also observed that, in the two cases of the modified adhesives subjected to seasoning, the value of the maximum force was similar to that obtained for the modified adhesive that was not subjected to seasoning.

On the basis of the results, it can be suspected that an increase in the modifier content will lead to higher elasticity of the adhesive. This applies primarily to Method II for preparation of modified adhesives. This may ensure greater beneficial behavior during the use of elements produced with these adhesive compositions and adhesive joints, i.e., better adaptation to changes in the shape of the elements and adhesive joints under the influence of external factors. Moreover, the increase in the quantity of the modifier decreases internal stresses in the cured adhesive composition, which contributes to better fuctioning of such a material in terms of the impact of external loads. The use of modifiers prevents material brittleness, which is a cause of destruction of such materials or adhesive joints caused by the impact of external stresses.

### 3.4. Microscopic Results

The effects of the adhesive compound preparation method applied (described in Table 2) and subjecting the adhesive compounds to seasoning (listed in Table 3) on the adhesive structure are given in Table 6.

As for Method I, it was observed that the biologically modified adhesive mixture of Epidian 53/PAC/1:1 applied to the micro slides exhibited changes at its edge at the 0.50% and 0.75% modifier contents compared to the results yielded in the control experiments. In Method II and the 0.25% modifier content, significant changes were observed in the specimen edge compared to those obtained in the 0% test. In the solution with the 0.50% and 1% modifier contents, the changes were smaller than those in the control experiment. In Method I, the changes were insignificant for the contents of 0.25% and 1.00%, and in Method II, the same was observed for the modifier content of 0.75%.

In Variant III (Method I and seasoning for 2 months), wherein the micro slides with the modifier were subjected to seasoning, the most significant changes, compared to the control experiment (no modifier applied), were observed for the adhesive specimens modified with the modifier contents of 0.50% and 0.75%.

## 4. Conclusions

The study investigating the properties of an adhesive modified with a lyophilized fungal formulation facilitated determination of certain mechanical properties of the modified and unmodified adhesives. The study involved a comparison of adhesive preparation methods and determination of the effect of seasoning on the mechanical properties of the tested specimens. The study also reported the results of a preliminary microscopic analysis, which will be extended in a planned experimental program.

It can be observed that both the applied methods of adhesive compound preparation and the applied modifier contents exerted a significant effect on the properties of the tested adhesive specimens. Tensile stresses and stresses at break were much higher in the adhesive specimens prepared with Method II. In Method I, an increase in the modifier content in the adhesive compound resulted in reduced stresses and less significant differences in specimen elongation at break. It can be claimed that the increased modifier content leads to a reduced elongation at break and reduced elasticity of the adhesive. In Method II, the elongation at break increased with increasing modifier content. The correlation ratio between these quantities was 0.95. It can therefore be suspected that an increase in the modifier content will lead to higher elasticity of the adhesive.

The seasoning of the modified adhesives did not have a negative effect on their mechanical properties. In turn, all the quantities tested in the unmodified adhesive decreased after seasoning. Although seasoning had a negative effect on the failure force, it led to a significant increase in the elasticity of the modified adhesive. In the majority of cases, the elongation at break of the modified adhesives subjected to seasoning increased by over 50% (even by over 70%) compared to the that of the modified adhesives that were not subjected to seasoning. In the modified adhesives, the elongation at break increased after the seasoning, compared to that of the unmodified adhesive cured for 7 days at ambient temperature.

The confocal microscopic images showed clear changes in the edges of the modified adhesive compounds, compared to the edges of the adhesives used in the control experiments (no biochemical modifiers applied). These changes were caused by both the method applied for preparation of the adhesive compound and specimen seasoning. As for Method II, it was observed that the specimen edges became smoother with an increase in the concentration of the fungal modifier. To sum up the results, it can be claimed that the high anti-oxidative potential of fungal modifiers as well as their qualitative composition (the content of phenolic compounds, low molecular weight of active proteins or carbohydrates) can significantly change the properties of the tested adhesive mixtures. Besides typical chemical reactions, probably typical mechanical changes in the adhesives directly affect their adhesive properties. However, the mechanisms of the described modifications require further research.

The results will be used for development of a technology for adhesive joints and designing a methodology of testing adhesive joints in various variants of working conditions. Currently, there are studies being conducted on the determination of the mechanical properties of cured modified epoxy adhesives subjected to thermal shock in established research cycles.

## Figures and Tables

**Table 1:** Modified adhesive compounds.

Components of the epoxy adhesive compound	Control test (g)	Test run 1 (g)	Test run 2 (g)	Test run 3 (g)	Test run 4 (g)
Epidian 53	50	50	50	50	50
PAC	50	50	50	50	50
Lyophilized preparation with a low molecular weight secondary metabolite subfraction	0.00	0.25	0.50	0.75	1.00

**Table 2:** Methods for preparation of the adhesive compound.

Denotation	Method description
Method I	The epoxy resin and curing agent were mixed with the fungal material in the desired concentration. The whole was mixed and used for production of the specimens of the cured adhesive and adhesive bonding of the sheets.
Method II	Resin was mixed with the mortar-grounded lyophilized material containing a low molecular weight secondary metabolite subfraction (LMS) obtained from idiophase fungal cultures of *Pycnoporus sanquineus* in the required concentration. After thorough mixing, the polyaminoamide curing agent was added, and the whole was mixed again.

**Table 3:** Conditions of specimen preparation for strength testing.

Variant	Method	Seasoning time	Seasoning conditions
Variant I	Method I	7 days	Temperature: 23 ± 2 °C
			Humidity: 23% ± 2%
Variant II	Method II	7 days	Temperature: 23 ± 2 °C
			Humidity: 23% ± 2%
Variant III	Method I	2 months	Temperature: 50 ± 1 °C
			Humidity: 50% ± 1%

**Table 4:** Tangent modulus *E_t_* of the modified adhesive specimens prepared with Methods I and II (mean values and standard deviation).

Amount of filler	Specimens prepared with Method I	Specimens prepared with Method II
	*E_t_*, MPa	*E_t_*, MPa
0.00%	419 ± 23	833 ± 24
0.25%	395 ± 27	743 ± 31
0.50%	429 ± 18	842 ± 23
0.75%	464 ± 16	839 ± 12
1.00%	379 ± 17	1024 ± 26

**Table 5:** Mechanical properties of the modified adhesive specimens prepared with Method I after the seasoning—Variant III (mean values).

Amount of filler	Specimens—Variant III					
	Fmax, N	*E_t_*, MPa	σ_γ_, MPa	σ_M_, MPa	ε_M_, %	σ_B_, MPa
0.00%	1055 ± 68	212 ± 12	8.8 ± 0.46	8.8 ± 0.18	10.0 ± 0.50	8.8 ± 0.22
0.25%	1400 ± 49	374 ± 18	-	11.4 ± 0.07	7.0 ± 0.18	11.4 ± 0.08
0.50%	1597 ± 59	222 ± 22	12.4 ± 0.35	12.4 ± 0.11	6.8 ± 0.12	10.7 ± 0.09
0.75%	1596 ± 87	251 ± 21	13.2 ± 0.18	13.2 ± 0.18	7.4 ± 0.16	10.7 ± 0.13
1.00%	1308 ± 12	237 ± 13	10.9 ± 0.12	10.9 ± 0.12	7.1 ± 0.08	7.4 ± 0.17

**Table 6:** Microscopic images of specimens prepared according to Methods I and II and subjected to seasoning (Variants I–III).

Amount of filler	Method I (Variant I)	Method II (Variant I)	Method I (Variant III)
0.00%	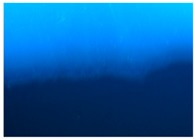	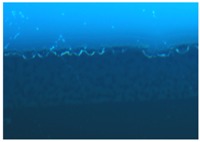	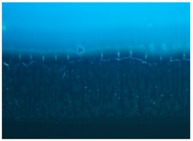
0.25%	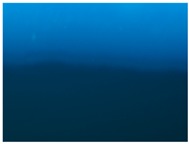	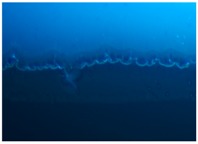	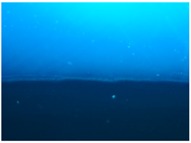
0.50%	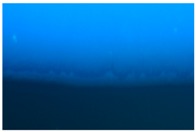	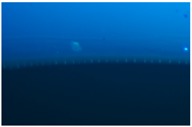	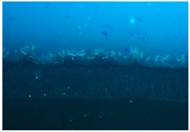
0.75%	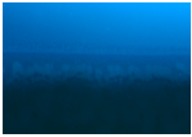	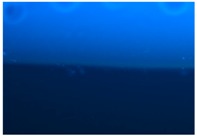	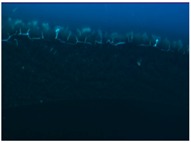
1.00%